# Palmar Cutaneous Erythematous Macules: A Case Report of an Atypical Presentation of Chilblain Lupus Erythematosus

**DOI:** 10.7759/cureus.103293

**Published:** 2026-02-09

**Authors:** Muhammad Israr ul Haq, Muhammad N Mahmood, Eunice Y Chow

**Affiliations:** 1 Medicine and Dentistry, University of Alberta, Edmonton, CAN; 2 Laboratory Medicine and Pathology, University of Alberta, Edmonton, CAN; 3 Medicine and Dermatology, University of Alberta, Edmonton, CAN

**Keywords:** chilblain lupus, chle, chronic cutaneous lupus erythematosus, cutaneous erythema, cutaneous lupus erythematosus, palmar cutaneous erythematous macules, palmar erythema, sle, systemic lupus erythema

## Abstract

Systemic lupus erythematosus (SLE) manifests with a broad spectrum of cutaneous manifestations, including chilblain lupus erythematosus (CHLE), which typically presents as cold-induced, painful acral lesions with specific dermatoscopic and histopathologic features. We describe an unusual case of a 32-year-old woman with established SLE and stage IV lupus nephritis who presented with a five-year history of asymptomatic, recurrent erythematous macules on the palms.

The patient reported no cold sensitivity, pain, or Raynaud’s phenomenon, which are hallmark features of classic CHLE. Physical examination revealed irregularly shaped pink macules and patches on the volar aspect of the fingers and thumb base and dorsal hands, sparing the palms. Dermoscopy showed well-demarcated, non-scaling pink macules with nonspecific white dots and sparing of palmar skin lines. Histopathological analysis demonstrated superficial and deep perivascular lymphocytic infiltrates with increased dermal mucin. Direct immunofluorescence revealed granular IgM and IgG deposits at the dermoepidermal junction, supporting a lupus-related process.

Although the patient met the histopathologic and minor clinical criteria for CHLE, the absence of cold triggers and pain suggests an atypical presentation of CHLE or a distinct variant of lupus-associated palmar macules. Differential diagnoses, including dermatomyositis and small-vessel vasculitis, were excluded through clinical and laboratory evaluations. This case highlights the clinical variability of cutaneous lupus and the diagnostic challenges posed by palmar lesions that do not strictly adhere to established diagnostic criteria for CHLE.

## Introduction

Systemic lupus erythematosus (SLE) is a chronic autoimmune disease that affects multiple organ systems, including the skin. Cutaneous lupus erythematosus (CLE) refers to lupus-specific skin involvement and is classically categorized into acute, subacute, and chronic forms of CLE [1,2]. Acute CLE typically presents in areas exposed to ultraviolet (UV) radiation with varying morphologies [1]. This consists of malar rash characterized by erythematous and edematous lesions, bullous lupus erythematosus presenting as vesiculobullous lesions, pruritic maculopapular eruptions, and Stevens-Johnson syndrome/toxic epidermal necrolysis (SJS/TEN)-like variant of CLE if severe enough [2]. Subacute CLE can present with papulosquamous or annular lesions in areas of UV radiation exposure or as a result of drug reaction [1]. Chronic CLE commonly presents as discoid lupus erythematosus, which is characterized by disk-shaped erythematous papules or plaques with peripheral hyperpigmentation and central hypopigmentation [2]. Less commonly, chronic CLE can present as chilblain lupus erythematosus (CHLE), which is a cutaneous manifestation of the underlying cold induced capillary inflammation causing erythematous to violaceous tender papules, plaques, and nodules that predominantly affect the fingers and toes [2]. 

In this case report, we describe a 32-year-old Asian woman who developed erythematous cutaneous palmar macules with a diagnosis of SLE. Considering the involvement of the bilateral palms and chronic presentation of these macules, we explored the possibility of chronic CLE subtype CHLE. However, her presentation did not completely fulfil the diagnostic criteria for CHLE and may represent an atypical presentation of CHLE. 

## Case presentation

A 32-year-old Asian woman with a known diagnosis of SLE and class IV lupus nephritis was referred to dermatology by her rheumatologist for assessment of a five-year waxing and waning history of asymptomatic erythematous macules on her hands. The patient denied cold exposure as a trigger for her lesions. There was no relationship with exposure to certain chemicals or other allergens. She had no smoking history. Review of systems was negative for myalgias, arthralgias, muscle weakness, pulmonary symptoms, photosensitivity, Raynaud’s phenomenon, alopecia, nasal or oral ulcerations, arthritis, pregnancy loss, or thrombosis. She did not have any eruptions elsewhere, including the toes. The onset of the red macules of the hands was approximately six months prior to the formal diagnosis of her SLE five years ago. 

The lesions on the hands initially improved with systemic therapy of prednisone 5 mg/day, mycophenolate mofetil (MMF) 1000 mg twice daily, and hydroxychloroquine 200 mg/day. She had used mometasone furoate 0.1% ointment once daily for two weeks to improve lesions with only mild intermittent flare-up. In the months prior to the dermatology referral, the hand lesions flared and no longer responded to the topical mometasone ointment. Coincidentally, her MMF had been discontinued a few months prior to this flare. 

**Figure 1:**
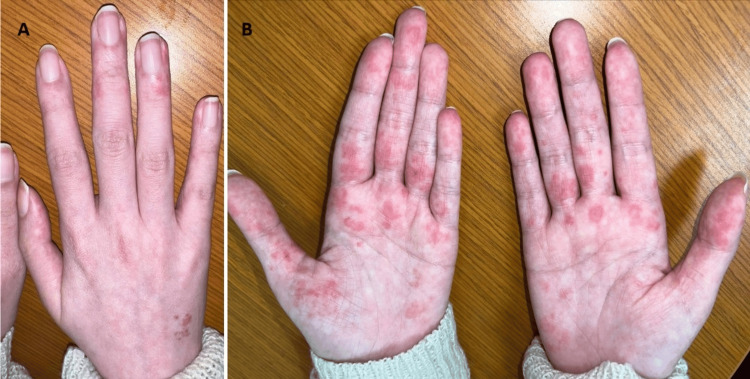
A: Clinical photo of the hands. A. The image of the right hand shows red papules on the dorsal hand, and erythematous macules of the lateral nail folds and proximal nail folds of the third and fourth digits; B. Clinical photo of the palms showing multiple erythematous macules and patches distributed over bilateral volar aspects of the phalanges, metacarpophalangeal joints, proximal and distal phalanges, and distal finger pads.

On physical examination of her hands, there were a few light pink, irregularly shaped macules measuring approximately 6-7mm on the right dorsal hand, and a few macules around the lateral nail folds of the third and fourth digits (Figure 1A). There was a more striking presentation with multiple erythematous macules and a few patches distributed over the base of the left thumb, the palmar aspect of the metacarpophalangeal joints, and the volar surfaces of the proximal and distal phalanges. The mid palm and the middle phalanges of the fingers were relatively spared (Figure 1B). There were no nail changes observed.

**Figure 2:**
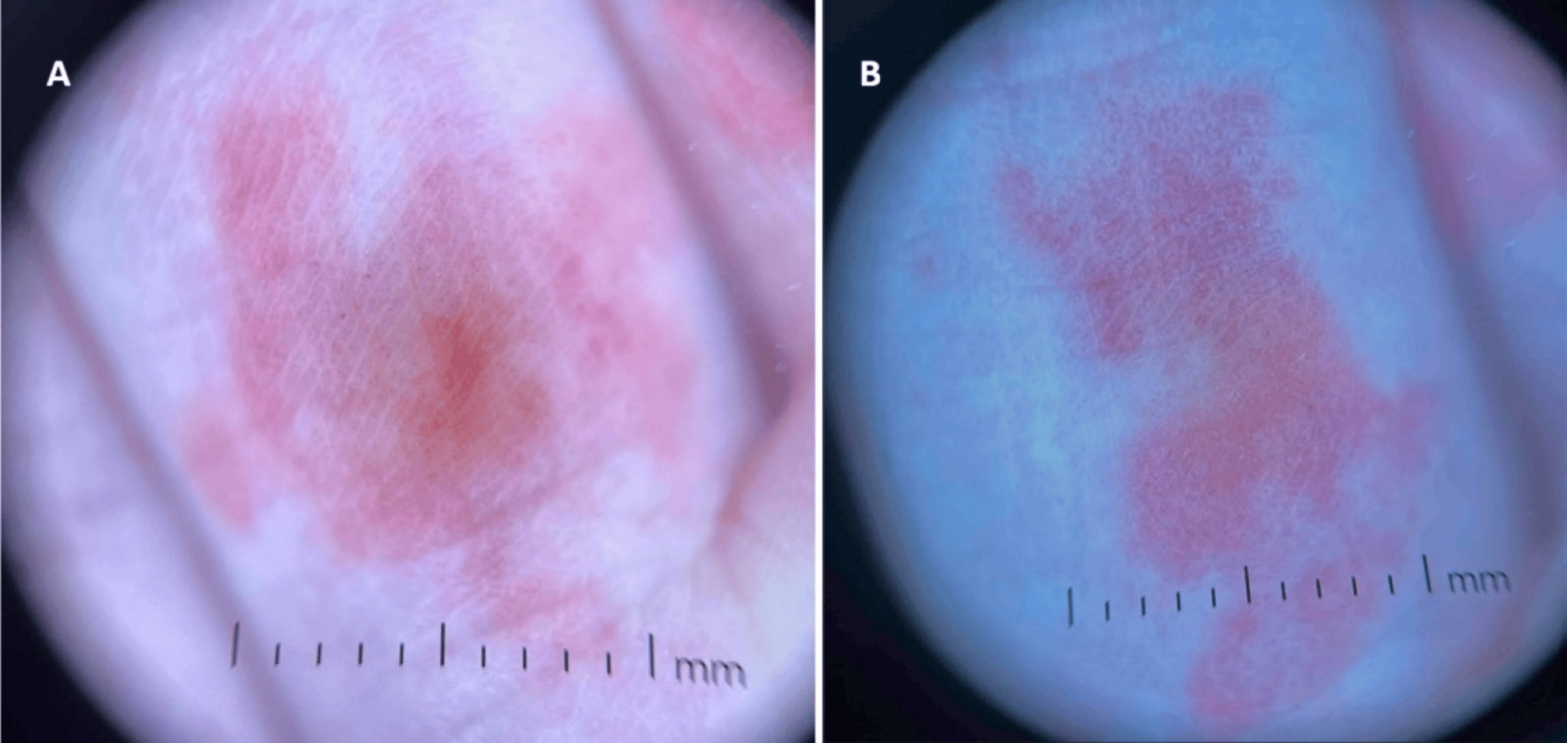
Dermoscopic images A. The image of the right hand ring finger showing a well-demarcated, irregularly shaped pink macule with a few dot vessels; B. Dermoscopic image of the left hand second digit volar proximal phalanx showing a well-demarcated, irregularly shaped pink macule with a few dot vessels, and sparing of the skin furrows and eccrine duct openings.

Dermoscopic examination of the affected areas demonstrated well-demarcated, non-scaling, irregularly shaped, pink macules with nonspecific white dots, and an apparent sparing of the skin lines and eccrine duct openings (Figures 2A, 2B). 

**Figure 3:**
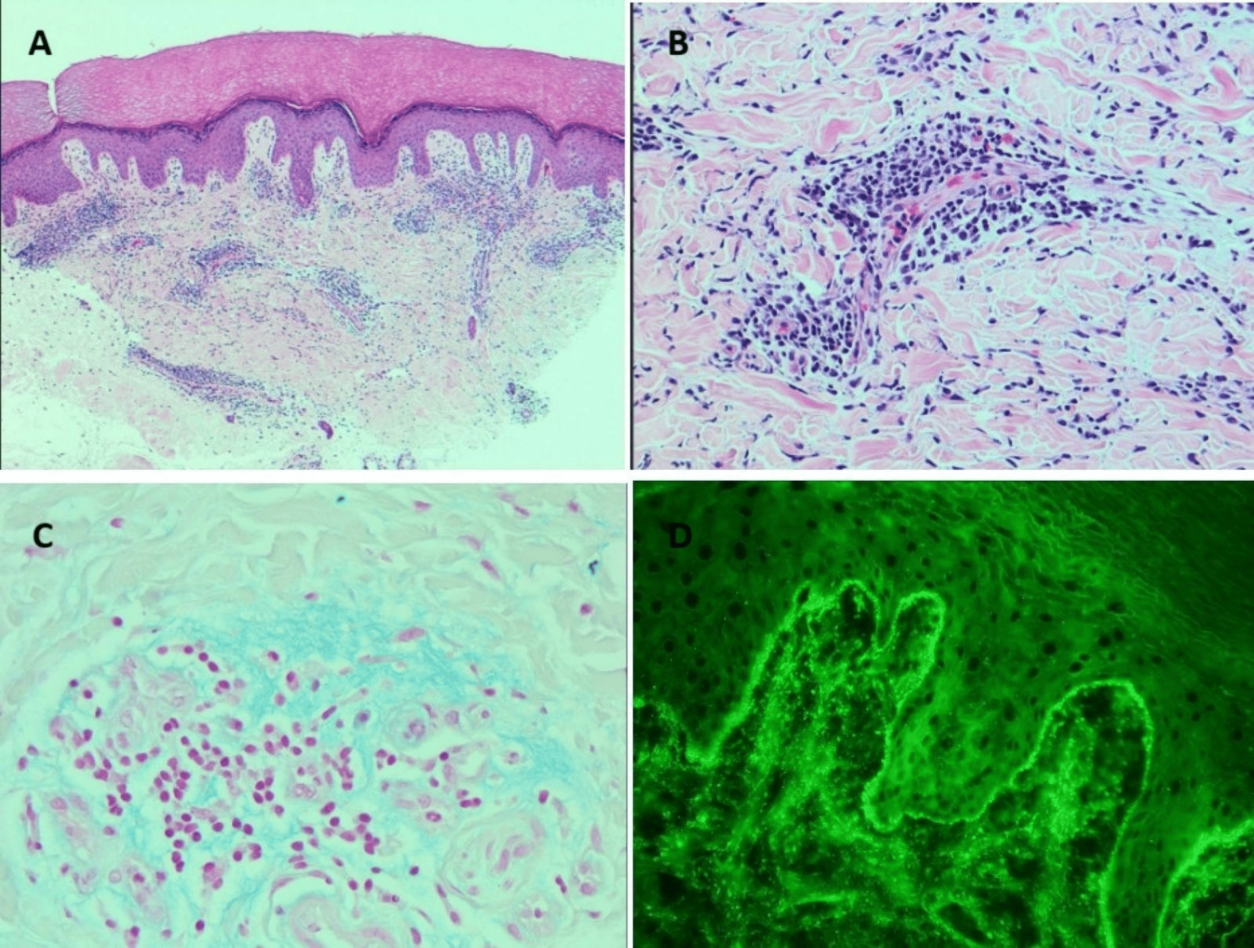
A: Punch biopsy. A. The image displays acral skin with superficial and deep lymphocytic infiltrate, epidermal spongiosis is not seen (H&E stain, x50); B: Perivascular lymphocytic infiltrate is noted in the reticular dermis, no eosinophils are seen (H&E stain, x200); C: Mucin deposition is noted in the reticular dermis (AB 2.5 stain (Alcian Blue, pH 2.5), x400); D: Direct immunofluorescence showing granular positivity at the dermoepidermal junction (IgM x200).

Multiple investigations were undertaken to assess the underlying etiology of this rash. Punch biopsy of the eruption on the hands was obtained, which showed superficial and deep perivascular lymphocytic infiltrate with dermal mucin (Figures 3A-3C). There was no significant epidermal spongiosis, eosinophilic infiltrate, or definitive interface dermatitis. Also, direct immunofluorescence studies showed granular IgM and IgG deposition at the dermoepidermal junction (Figure 3D). 

Given the palmar presentation of the rash similar to inverse Gottrón’s papules, screening investigations for dermatomyositis were undertaken. The patient did not endorse any proximal muscle weakness, and her creatinine kinase levels were within normal range. Her autoimmune myopathy panel workup for dermatomyositis was largely negative, with the exception of having medium positive anti-Ro52 and weak positive anti-Ku Helicase. These antibodies can also be detected in SLE. Thus, her rheumatologist did not consider these positive antibodies to be indicative of an autoimmune inflammatory muscle disease, especially in the absence of clinical symptoms of a muscle disease.

We also noted that at the time of the patient’s cutaneous symptoms flare up, she had an increase in her anti-double-stranded DNA (anti-dsDNA) antibody level to 53 kIU/L (kilo international units per litre) from a prior level of 5 kIU/L. The anti-dsDNA levels gradually stabilized and decreased to 12 kIU/L as the severity of her cutaneous macules improved. Her extractable nuclear antigens (ENAs) screen was positive with elevated levels of Sjögren's syndrome-related antigen A (SSA)-52 antibody (1.5 antibody index (AI), elevated titers of SSA-60 (>8.0 AI), anti-Smith (Sm) antibodies (>8.0 AI), anti-Smith/ribonucleoprotein (Sm/RNP) antibodies (8.0 AI), anti-U1 ribonucleoprotein A (RNP-A) antibodies (>8.0 AI) and anti-chromatin antibodies (5.6 AI). Her cryoglobulin and cryofibrinogen testing were negative. 

**Figure 4:**
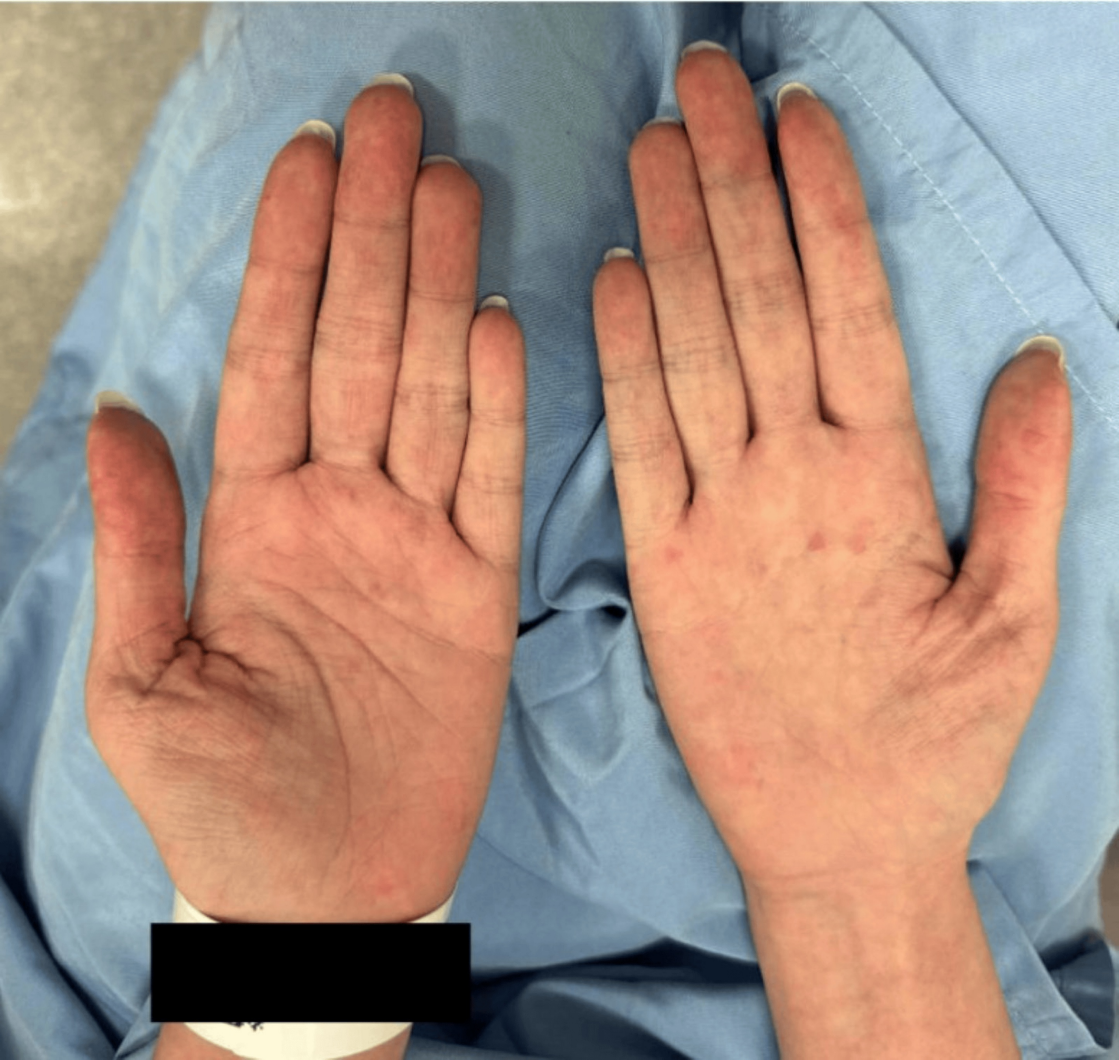
Clinical image of her palms showing fewer erythematous macules.

Her hand lesions continued to persist despite being on hydroxychloroquine 200 mg daily. However, the severity of her lesions subsided significantly with the application of fluocinonide 0.05% ointment applied twice daily for three weeks (one week off) for three months. At this time, she uses the topical steroid intermittently when she develops mild flares of her hand lesions (Figure 4). 

## Discussion

Chilblain lupus erythematosus (CHLE) is typically diagnosed when both major criteria and at least one minor criterion are met. The major criteria include acral skin lesions triggered or worsened by cold exposure and evidence of cutaneous lupus erythematosus as determined by histopathologic examination or immunofluorescence study [3,4]. The minor criteria include the coexistence of systemic or discoid lupus, improvement with lupus-directed therapy, and negative testing for cryoglobulin and cold agglutinin [3,4]. 

Our patient did not report a cold-related pattern, which makes it difficult to satisfy the first major criterion. However, her biopsy showed perivascular lymphocytic infiltrates, increased dermal mucin, and granular IgM and IgG deposits at the dermoepidermal junction. These histopathological findings overlap with descriptions of CHLE, which often show similar lymphocytic infiltrates, mucin deposition, and immune complex deposition [4]. She met all of the minor criteria, including established SLE diagnosis, improvement of the lesions with anti-lupus treatment, and negative cryoglobulin and cryofibrinogen testing. The absence of one of the major criteria of cold sensitivity, along with a lack of pain, which is commonly reported in CHLE, makes the CHLE diagnosis less certain [3,4]. 

Dermoscopic information for palmar lesions in lupus is limited. Żychowska et al. described dermoscopic patterns in chronic cutaneous lupus across several anatomic sites, including a small subset of CHLE lesions [5]. Common dermoscopic features of CHLE reported included a pink-red background, dotted vessels, linear, branched or curved vessels, scaling, and red structureless areas [5]. Our patient had a pink background and dotted vessels, but she lacked scaling. The sparing of palmar furrows was not noted in their cohort, which we noted in our patient. Therefore, the dermoscopic findings only partially align with previously reported CHLE features.

Other lupus-associated palmar macules have been described. Richter et al. reported palmar lesions in the setting of small-vessel vasculitis in systemic lupus, including erythematous macules on volar pressure points [6]. However, our patient’s biopsy did not show features of vasculitis. In a cohort of 50 patients with digital lesions in systemic lupus, Bouaziz et al. found a range of diagnoses, including acute, subacute, and discoid cutaneous lupus, urticarial vasculitis, and CHLE [7]. They emphasized that clinical appearances are variable and that histopathology is often needed to classify these lesions. In comparing their descriptions, CHLE remains the closest match among the established categories for our patient. Nonetheless, our patient does not fully meet the expected clinical pattern for CHLE.

We also considered dermatomyositis with inverse Gottrón’s papules, given the distribution of the macules [8]. Histologic findings in dermatomyositis can include perivascular inflammation, vacuolar interface changes, increased mucin, telangiectasias, and hyperkeratosis [9]. Our patient shared only the mucin deposition and perivascular infiltrates. She had no muscle weakness, systemic symptoms characteristic of dermatomyositis, or elevations in creatine kinase. Anti-Ro52 and anti-Ku antibodies were present; however, they are not specific to dermatomyositis and have been reported in systemic lupus [10,11]. Taken together, these findings suggest dermatomyositis is unlikely.

Given the incomplete fulfilment of the CHLE criteria and the lack of alignment with other lupus-associated or dermatomyositis-associated digital lesions, we interpret this presentation as most consistent with an atypical form of chilblain lupus erythematosus, despite the absence of cold sensitivity and pain. The histologic evidence of cutaneous lupus supports overlap with reported CHLE features, fulfilment of all minor criteria, and response to lupus-directed therapy support this classification. Although this presentation does not fully match the expected clinical pattern of CHLE, alternative lupus-associated palmar eruptions appear less consistent with the overall clinicopathologic findings.

## Conclusions

This case describes a patient with SLE who developed recurrent, asymptomatic erythematous macules on the volar hands that we classify as an atypical presentation of CHLE. Although the presentation did not fulfil all established diagnostic criteria, particularly with respect to cold sensitivity and typical distribution, the overall clinicopathologic context supports inclusion within the CHLE spectrum. This case highlights the phenotypic variability of cutaneous lupus and underscores the importance of integrating clinical features with histopathologic findings when lesions fall outside classic patterns.
